# The Kipoi repository accelerates community exchange and reuse of predictive models for genomics

**DOI:** 10.1038/s41587-019-0140-0

**Published:** 2019-05-28

**Authors:** Žiga Avsec, Roman Kreuzhuber, Johnny Israeli, Nancy Xu, Jun Cheng, Avanti Shrikumar, Abhimanyu Banerjee, Daniel S. Kim, Thorsten Beier, Lara Urban, Anshul Kundaje, Oliver Stegle, Julien Gagneur

**Affiliations:** 1 Department of Informatics, Technical University of Munich, Garching, Germany; 2 Graduate School of Quantitative Biosciences (QBM), Ludwig‐Maximilians‐Universität München, Munich, Germany; 3 Department of Haematology, University of Cambridge, Cambridge, UK; 4 European Molecular Biology Laboratory, European Bioinformatics Institute, Hinxton, UK; 5 Biophysics Program, Stanford University, Stanford, CA USA; 6 Department of Computer Science, Stanford University, Stanford, CA USA; 7 Physics Department, Stanford University, Stanford, CA USA; 8 Biomedical Informatics Program, Stanford University, Stanford, CA USA; 9 Division for Computational Genomics & Systems Genetics, German Cancer Research Center, Heidelberg, Germany; 10 European Molecular Biology Laboratory, Genome Biology Unit, Heidelberg, Germany; 11 Department of Genetics, Stanford University, Stanford, CA USA

**Keywords:** Computational biology and bioinformatics, Genome informatics, Machine learning

**To the Editor** — Advances in machine learning, coupled with rapidly growing genome sequencing and molecular profiling datasets, are catalyzing progress in genomics^1^. In particular, predictive machine learning models, which are mathematical functions trained to map input data to output values, have found widespread usage. Prominent examples include calling variants from whole-genome sequencing data^2,3^, estimating CRISPR guide activity^4,5^ and predicting molecular phenotypes, including transcription factor binding, chromatin accessibility and splicing efficiency, from DNA sequence^1,6–11^. Once trained, these models can be probed in silico to infer quantitative relationships between diverse genomic data modalities, enabling several key applications such as the interpretation of functional genetic variants and rational design of synthetic genes.

However, despite the pivotal importance of predictive models in genomics, it is surprisingly difficult to share and exchange models effectively. In particular, there is no established standard for depositing and sharing trained models. This lack is in stark contrast to bioinformatics software and workflows, which are commonly shared through general-purpose software platforms such as the highly successful Bioconductor project^12^. Similarly, there exist platforms to share genomic raw data, including Gene Expression Omnibus (https://www.ncbi.nlm.nih.gov/geo/), ArrayExpress (https://www.ebi.ac.uk/arrayexpress) and the European Nucleotide Archive (https://www.ebi.ac.uk/ena). In contrast, trained genomics models are made available via scattered channels, including code repositories, supplementary material of articles and author-maintained web pages. The lack of a standardized framework for sharing trained models in genomics hampers not only the effective use of these models—and in particular their application to new data—but also the use of existing models as building blocks to solve more complex tasks.

Repositories of trained models (Supplementary Table 1), which are routinely used for benchmarking and as a starting point to rapidly develop new models in computer vision and natural language processing, hold the promise to overcome these challenges. However, although generic model repositories exist, these are geared toward a technical audience of machine-learning experts. In contrast, a repository of trained models for genomics needs to be easy to use and deliver robust and well-documented software to enable application by practitioners who do not have expert knowledge in machine learning. A second challenge is the heterogeneity of machine-learning frameworks that are used, including Keras (https://keras.io), Tensorflow (https://tensorflow.org), PyTorch (https://pytorch.org) and custom model code, which is not addressed by current repositories. Furthermore, a model repository for genomics requires additional developments to support data formats and necessary processing steps for data produced by different genomics technologies. Finally, applications in genomics impose specific requirements on the interpretability of models, for example, to understand changes in phenotype for different DNA sequence inputs.

Here, we present Kipoi (Greek for ‘gardens’, pronounced ‘kípi’), an open science initiative to foster sharing and reuse of trained models in genomics. Already, the Kipoi repository (Fig. 1, middle) offers more than 2,000 individual trained models from 22 distinct studies that cover key predictive tasks in genomics, including the prediction of chromatin accessibility, transcription factor binding, and alternative splicing from DNA sequence. Kipoi is accessible via GitHub and as web resource (https://kipoi.org), providing a browsable interface to explore and search models for specific tasks.

**Fig. 1:**
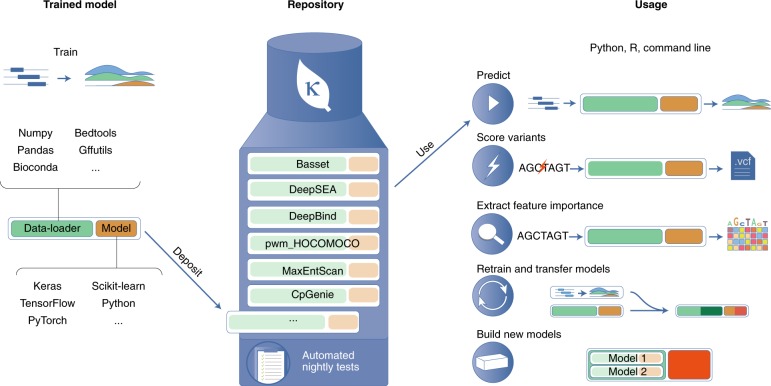
Overview of Kipoi. From left to right: at its core, Kipoi defines a programmatic standard for data-loaders and predictive models. Data-loaders translate genomics data into numeric representations that can be used by machine learning models. Kipoi models can be implemented using a broad range of machine-learning frameworks. The Kipoi repository allows users to store and retrieve trained models, together with associated data-loaders. Kipoi models are automatically versioned, nightly tested and systematically documented with examples of their use. Kipoi models can be accessed through unified interfaces from python, R and the command line. All models and their software dependencies can be installed in a fully automatic manner. Kipoi streamlines the application of trained models to make predictions on new data, to score variants stored in the standard variant call format (.vcf) file format, and to assess the effect of variation in the input to model predictions (feature importance score). Moreover, Kipoi models can be adapted to new tasks either by retraining them or by building new composite models that combine existing ones. Newly defined models can be deposited in the repository.

One of the core innovations of Kipoi includes standardized data handling (data-loaders) (Fig. 1, left). Data-loaders abstract and unify the preprocessing of data stored in bioinformatics file formats, yielding numerical representations that can be used as model inputs. Kipoi defines an application programming interface (API; Fig. 1, right; i.e., a standard way for software components to communicate with Kipoi models), which allows programmers to interchangeably use Kipoi models in their software with minimal coding effort. The Kipoi API is accessible from python and R, two of the most popular programing languages in computational biology. The API can also be accessed via the command line, which facilitates the integration of Kipoi models into bioinformatics workflows.

To ensure sustainability of trained models and to facilitate their dissemination, Kipoi builds on and interoperates with a range of software development technologies and standards. The model descriptions and the code of Kipoi are stored in GitHub repositories, providing issue tracking to facilitate transparent and rapid user–developer iterations. Moreover, by building on GitHub, we track and index both the Kipoi core code and contributed models, which facilitates reproducible research. The Kipoi model definition describes the model inputs and outputs, specifies the data-loader and required dependencies, and provides information about the source publication or the distribution license. Kipoiseq (https://github.com/kipoi/kipoiseq/), a companion python package, provides ready-to-use data-loaders for canonical sequence-related bioinformatics data types. Model parameters or other non-source files are hosted on Zenodo or Figshare—data repositories that offer a digital object identifier (DOI) and ensure long-term data access. Kipoi enables seamless installation of models and their software dependencies independently of the programming language of the model (by providing containers or using Conda and pip package managers, hence leveraging the Bioconda distribution^13^; Supplementary Methods). New models can be contributed using a simple, well-documented workflow (Supplementary Methods). Moreover, all models are subjected to nightly tests using a continuous integration service (CircleCI), thereby ensuring that all models are executable and yield reproducible outputs on test datasets^14^. Below, we illustrate usage of Kipoi through five relevant use cases and make the code available for each of them.

## Benchmarking alternative models for predicting transcription factor binding

**Fig. 2:**
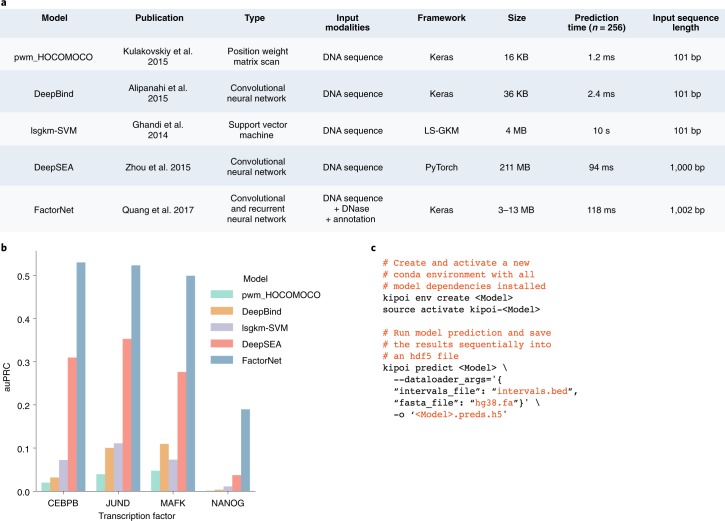
Using Kipoi to apply and benchmark alternative models for transcription factor binding prediction. **a**, Five models for predicting transcription factor binding based on alternative modeling paradigms: first, position weight matrices provided by the HOCOMOCO database^28^; second, lsgkm-SVM^15^, a support vector machine classifier; third, the convolutional neural network DeepBind^6^; fourth, the multi-task convolutional neural network DeepSEA; and finally, FactorNet, a multimodal deep neural network with convolutional and recurrent layers that further integrates chromatin accessibility profile and genomic annotation features. Models differ by both the size of genomic input sequence (DeepSEA^7^ and FactorNET^8^ consider ~1 kb, whereas other models are based on ~100 bp sequence inputs) and the parametrization complexity, with the total size of stored model parameters ranging from 16 kB (pwm_HOCOMOCO) to 211 MB (DeepSEA). **b**, Performance of the models in **a** for predicting ChIP-seq peaks of four transcription factors on held-out data (chromosome 8), quantified using the area under the precision recall curve (auPRC). More complex models yield more accurate predictions than the simpler models such as the commonly used position weight matrices. **c**, Example use of Kipoi from the command line to install software dependencies, download the model, extract and preprocess the data, and write predictions to a new file. Results as shown in **b** can be obtained for all Kipoi models listed in **a** using these generic commands by varying the placeholder <Model>.

Practitioners are often faced with the choice between multiple predictive models. Identifying the most appropriate model often requires them to perform a benchmark on data relevant to the application. Access to a wide range of models through a common API facilitates the systematic comparison of models. To illustrate this use case, we benchmarked five models for predicting genomic binding sites of transcription factors (Fig. 2a). These models span different modeling paradigms, including methods based on classical position weight matrices, gapped *k*-mer support vector machines (lsgkm-SVM^15^) and deep learning (DeepBind^6^, DeepSEA^7^ and FactorNet^8^). The models were assessed for distinguishing bound from unbound regions, where bound regions were defined as high-confidence binding events in chromatin immunoprecipitation sequencing (ChIP-seq) experiments for four transcription factors in different cell lines: CEBPB in HeLa-S3, JUND in HepG2, MAFK in K562 and NANOG in H1-hESC (Supplementary Methods). With the exception of lsgkm-SVM (Supplementary Table 1), all Kipoi implementations of the considered models are based on implementations provided by the respective publications and were trained by the original authors. The performance of all models was assessed on chromosome 8, which was not used to train any of the considered models.

Position weight matrix models performed poorly across all transcription factors (Fig. 2b), likely owing to their inability to account for additional sequence features, such as motifs of other cooperating and competing transcription factors. More complex models (for example, DeepSEA and FactorNet) consistently outperformed simpler ones (for example, DeepBind and lsgkm-SVM). FactorNet consistently yielded the most accurate predictions, most likely because the model combines sequence and DNA accessibility information (Fig. 2b and Supplementary Fig. 1).

In this example, Kipoi greatly simplifies an otherwise cumbersome task. The considered models are implemented with different software frameworks (Fig. 2a), require different input file formats, and return predictions in different output formats. Furthermore, installing and validating the appropriate software dependencies for each model is difficult and time consuming when done manually. With Kipoi, the entire procedure of installing and executing a model reduces to executing three simple commands (Fig. 2c). As these three commands are common to all models and the predictions are stored in a common format, the benchmark can be very simply scripted with workflow management tools (Supplementary Methods).

## Improving predictive models of chromatin accessibility via transfer learning

Training new models can be time consuming and requires large training datasets. One way this can be facilitated is via transfer learning (i.e., reusing models trained on one prediction task to initialize a new model for a different but related task)^16^. Transfer learning typically enables more rapid training, reduces the required amount of data for training and improves the predictive performance compared with models trained from scratch. Deep neural networks are well suited to transfer learning. They consist of successive layers that transform input data into increasingly abstract representations. Most of the low-level abstractions—for instance, edge detection for images or transcription factor motifs in genomics—turn out to be common to multiple prediction tasks. Consequently, it is often sufficient to train only the more abstract layers when transferring such models to solve a new task. Transfer learning of deep neural networks has been successfully applied across multiple domains, including biological imaging^17^, natural language processing^18^ and genomics^19^.

**Fig. 3:**
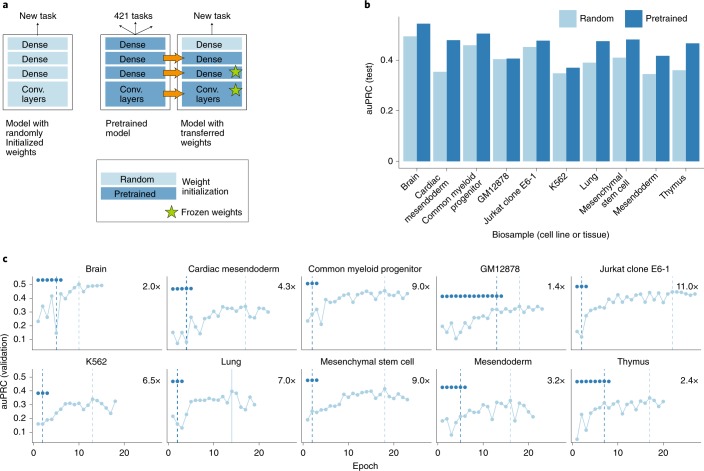
Using Kipoi for adapting existing models to new tasks (transfer learning). **a**, Architecture of alternative models for predicting chromatin accessibility from DNA sequence. Model parameters were either randomly initialized (left) or transferred from an existing neural network pretrained on 421 other biosamples (cell lines or tissues, right). **b**, Predictive performance measured using the area under the precision recall curve (auPRC), comparing randomly initialized (light blue) versus pretrained (dark blue) models. Shown is the performance on held-out data (chromosomes 1, 8 and 21) for 10 biosamples that were not used during pretraining. **c**, Training curves, showing the auPRC on the validation data (chromosome 9) as a function of the training epoch. The dashed vertical line denotes the training epoch at which the model training was completed. Pretrained models required fewer training epochs than randomly initialized models and achieved more accurate predictions.

Here we revisit a transfer learning example in genomics^19^, predicting chromatin accessibility profiles for 431 biosamples (cell lines or tissues; Supplementary Methods). Initially, we trained a genome-wide multi-task model to predict chromatin accessibility for 421 biosamples (tasks), while holding out 10 biosamples. For the 10 held-out biosamples, we trained single-task models, one per biosample, transferring all model parameters but the final layer (Fig. 3a). The final two layers of this model were then retrained for each task while keeping the remaining model parameters fixed. For comparison, we also considered single-task models with randomly initialized parameters but otherwise identical architecture. Models initialized with transferred model parameters yielded improved predictive accuracy for all biosamples, with a 15.1% larger area under the precision recall curve on average, compared to conventional training using randomly initialized parameters (Fig. 3b). Transfer learning also greatly reduced the required training time, from over a day to 7 h on average (5.4 epochs versus 17.3 epochs on average; Fig. 3c).

Kipoi promotes transfer learning in three ways. First, it provides access to a comprehensive collection of state-of-the-art models in genomics. Transfer learning works better if the tackled task is similar to the original task of the pretrained model^16^. Kipoi allows users to efficiently access a large collection of trained models, which can be browsed by name, tag or framework, thus facilitating the identification of models trained for related tasks. Second, each model is easily installable and comes with a tested data-loader. Most of the data-loaders can be directly used to retrain models. Third, for neural network models, Kipoi offers a command to return and store the activation of a desired intermediate layer rather than the final, prediction layer. Using these precomputed intermediate activations can substantially speed the training of the transferred model. A second advantage of storing the intermediate activation is that any framework can be used to train the top layers. Altogether, leveraging pretrained models—in particular, deep neural networks that have been trained on large datasets with a substantial investment in computing time—allows researchers to train more accurate models on smaller datasets while saving time and computing costs.

## Predicting the molecular effects of genetic variants using interpretation plugins

**Fig. 4:**
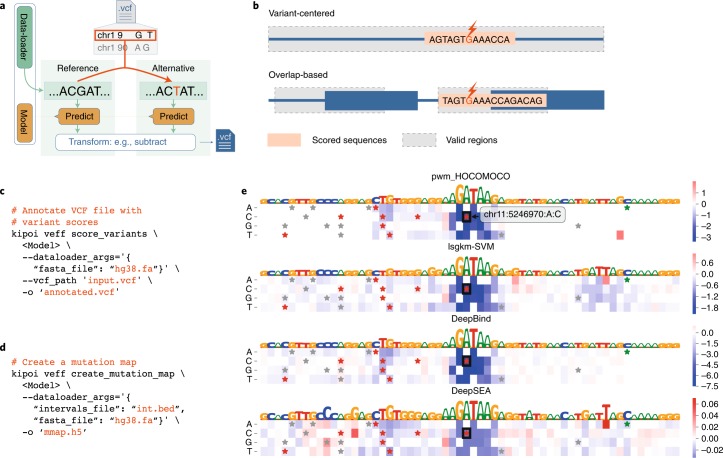
Variant effect prediction and feature importance scores. **a**, Schema of variant effect prediction using in silico mutagenesis. Model predictions calculated for the reference allele and the alternative allele are contrasted and written into an annotated copy of the input variant call format file (.vcf). **b**, Kipoi uniformly supports variant effect prediction for models that can make predictions anywhere in the genome (top) and also for models that can make predictions only on predefined regions such as exon boundaries (bottom). **c**, Generic command for variant effect prediction. **d**, Generic command to compute the importance scores using in silico mutagenesis. **e**, Feature importance scores visualized as a mutation map (heat map: blue, negative effect; red, positive effect) for variant rs35703285 and the predicted GATA2 binding difference between alleles for four different models. The black boxes in the mutation maps highlight the position and the alternative allele of the respective variant. Stars highlight variants annotated in the human variant database ClinVar, with red indicating likely pathogenic; green, likely benign; gray, uncertain, conflicting significance, and any other type.

One important application of trained models in genomics, with translational relevance in human genetics and cancer research, is to predict the effects of genetic variants on molecular phenotypes^7,20^. Individually, variant effect prediction has been implemented by a subset of published sequence-based predictive models, such as DeepBind^6^, DeepSEA^7^ and CpGenie^20^. Kipoi provides a generic and standardized implementation of variant effect prediction as a plugin, which allows for annotating variants obtained from the variant call format (.vcf) files in conjunction with DNA sequence-based models (98% of models in the Kipoi repository). The variant effect prediction plugin performs in silico mutagenesis by contrasting model predictions for the reference allele and for the alternative allele (Fig. 4a,b). If the model can be applied across the entire genome, such as in chromatin accessibility models, sequences centered on the queried variants are extracted (top row, Fig. 4b). If instead the model can only be applied to regions anchored at specific genomic locations, such as in splicing models at intron–exon junctions, only sequences extracted from valid regions that overlap with the variants of interest are used (bottom row, Fig. 4b). Kipoi provides a single command handling both scenarios (Fig. 4c). Altogether, the variant effect prediction plugin allows integrating a broad range of genomics predictive models into personal genome annotation workflows, and it can be readily extended to newly added models.

To inspect genomic regions containing the variant in higher detail, variant effect predictions for all possible single nucleotide variants in the sequence can be computed using a single command (Fig. 4d) and visualized as a mutation map (Fig. 4e). This helps to assess the predicted impact of a variant of interest in the context of other possible variants in the genomic region and may help pinpoint affected *cis*-regulatory elements. For example, the mutation maps for transcription factor binding sites of GATA2 show that the first four models from Fig. 2 agree on the effect of the variant rs35703285. Interestingly, the three most complex models (lsgkm-SVM, DeepBind and DeepSEA) predict effects of similar strength further away from the core motifs. This likely reflects that these models capture further regulatory sequences beyond the core motif. The variant rs35703285 has previously been classified as pathogenic in the ClinVar dataset and is linked to β-thalassemia (MedGen: C0005283), a disease that reduces synthesis of the hemoglobin subunit β (hemoglobin β chain) and results in microcytic, hypochromic anemia. The mutation map indicates that similar loss of GATA2 binding can be expected from other variants in the region.

In addition to in silico mutagenesis, which only applies to sequences, Kipoi provides a plugin that can evaluate the influence for any type of input on model prediction by implementing feature importance algorithms, including saliency maps^21^ and DeepLIFT^22^. These algorithms can offer complementary insights and are computationally more efficient than in silico mutagenesis.

## Predicting pathogenic splice variants by combining models

**Fig. 5:**
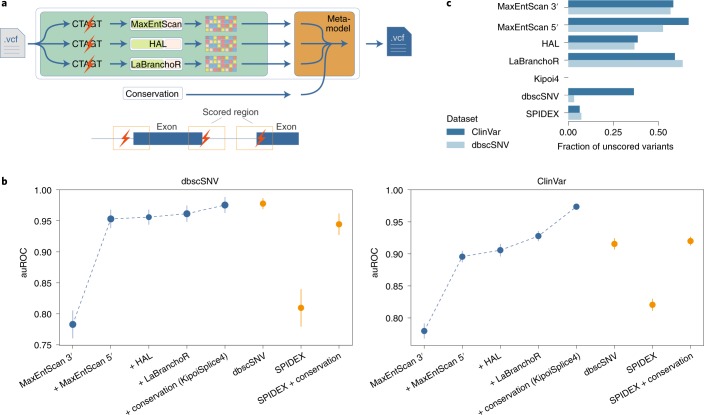
Composite models using Kipoi for improved pathogenic splice variant scoring. **a**, Illustration of composite modeling for mRNA splicing. A model trained to distinguish pathogenic from benign splicing region variants is easily constructed by combining Kipoi models for complementary aspects of splicing regulation (MaxEntScan 3′ models the acceptor site, MaxEntScan 5′ and HAL model the donor site, LaBranchoR models the branchpoint) and phylogenetic conservation. These variant scores are combined by logistic regression to predict the variant pathogenicity (orange box). **b**, Different versions of the ensemble model were trained and evaluated in tenfold cross-validation for the dbscSNV and ClinVar datasets (Supplementary Methods). The four leftmost models are incrementally added to the composite model in chronological order of their publication: the leftmost point only uses information from the MaxEntScan 3′ model, while “+ conservation (KipoiSplice4)” uses all four models and phylogenetic conservation. These performances were compared to a logistic regression model using state-of-the-art splicing variant effect predictors (SPIDEX, SPIDEX + conservation, dbscSNV). KipoiSplice4 achieves state-of-the-art performance on the dbscSNV dataset and outperforms alternative models on ClinVar, which contains a broader range of variants. auROC, area under the receiver operating characteristics curve. **c**, Fraction of unscored variants for different models in the dbscSNV and ClinVar datasets.

State-of-the-art models performing variant effect prediction frequently combine scores from multiple models. The advantage is twofold. First, combined scores can cover multiple biological processes. Second, combined scores are more robust because they average out conflicting predictions of individual models. Combining models or scores can be easily done in Kipoi by leveraging the standardization and modularity of models in combination with the variant effect prediction plugin introduced above. As a proof of concept, we used Kipoi to define a pathogenicity score of variants located near splice sites by integrating four predictive models covering complementary aspects of splicing (Fig. 5a) into a single composite model.

Splicing defects are one of the most frequent causes of genetic disease. In the first step of splicing, the donor site is attacked by an intronic adenosine to form a branchpoint. In the second step, the acceptor site is cleaved and spliced (i.e., joined) to the 3′ end of the donor site. To cover variants possibly affecting splicing through different mechanisms, we considered four complementary models trained on different types of data. The first two models were 5′ and 3′ MaxEntScan, which are based on a probabilistic model that scores donor and acceptor site regions and was trained on splice sites with cDNA support^9^; the third model was HAL, a *k*-mer based linear regression model scoring donor site regions that was trained on a massively parallel reporter assay in which hundreds of thousands of random sequences probed the donor site sequence space^10^; and the fourth model was LaBranchoR, a deep-learning model scoring the region upstream of the acceptor site for possible branchpoint locations that was trained from experimentally mapped branchpoints^11,23^.

Although MaxEntScan can be easily applied to score genetic variants provided in VCF files through Ensembl’s variant effect predictor plugin^24^, HAL and LaBranchoR do not offer this functionality out of the box. Using Kipoi’s API, the variant effect prediction is standardized for all these models (Fig. 5a). We built a new Kipoi model, KipoiSplice4, which is a logistic regression model based on variant effect predictions of these four Kipoi models and phylogenetic conservation scores (Supplementary Methods and Fig. 5a). This combined model was trained on two different datasets of splice variants classified either as pathogenic or benign (dbscSNV and ClinVar; Supplementary Methods).

To illustrate the benefit of integrating multiple models, we incrementally added the four splicing models in the chronological order of model publication. With an increasing number of models, the performance increased in both dbscSNV and ClinVar datasets (Fig. 5b, left four methods). Next we evaluated the model performance against two state-of-the-art splicing scores: another integrative approach that predicts pathogenic splicing-affecting variants, dbscSNV^25^, and SPIDEX^26^. For a fair comparison, we furthermore trained a score combining SPIDEX and phylogenetic conservation on each dataset, which reached the same performance as the dbscSNV model on ClinVar. While the performance of KipoiSplice4 is similar to that of dbscSNV for the dbscSNV dataset, KipoiSplice4 outperforms all other methods on the ClinVar dataset. One reason for the better performance of KipoiSplice4 is that it scores more variants (Fig. 5c). Neither SPIDEX nor dbscSNV explicitly models the splicing branchpoint, while KipoiSplice4 does so using LaBranchoR.

By wrapping the individual models into a data-loader, we made the ensemble model KipoiSplice4 available in Kipoi. KipoiSplice4 can hence be executed on demand to de novo predict effects of variants in splice sites. Altogether, by wrapping existing splice models into Kipoi, and thereby leveraging the out-of-the-box variant effect prediction, we developed a state-of-the-art model for scoring the pathogenicity of splicing variants. Additionally, with new splicing models and more extensive training datasets of better quality being published, the ensemble model can be easily and transparently improved.

## A unique resource

We have introduced a repository and programmatic standard for sharing and reuse of trained models in genomics, thereby addressing an unmet need. The Kipoi model repository is dedicated to trained models with applications in genomics in the broad sense. Specifically, we request at least one input data modality that can be derived either from DNA sequence (which includes amino acid sequence) or from an -omics assay, such as ChIP-seq or protein mass spectrometry. By providing a unified interface to models, automated installation, and nightly tests, Kipoi streamlines the application of trained models, overcomes the technical hurdles of their deployment, improves their dissemination, and ultimately facilitates reproducible research. The use cases we have presented demonstrate that Kipoi greatly facilitates the execution and comparison of alternative models for the same task, standardizes their use to functionally interpret genetic variants, and facilitates the development of new models based on existing ones, either by means of transfer learning or by model combination.

The dissemination and sharing of trained models offers key advantages over either sharing precomputed predictions or sharing code for users to train models from scratch. Precomputed predictions are limited to a narrow set of predefined input data. In particular, for DNA sequence variations, the combinatorial growth of possible sequence variants renders this approach impractical in terms of storage and compute requirements. For example, storing variant effect predictions is technically impossible even for relatively short (<10 bp) indels. Conversely, retraining models from scratch is technically challenging and requires access to potentially large training dataset, as well as suitable computational resources. Trained machine learning models can be regarded as functions encoding data distributions^27^. We anticipate the relevance of sharing trained models increasing as larger datasets are becoming available, with repositories such as Kipoi filling an important gap between code repositories and data archives.

Transfer learning appears to be a promising avenue for training models when data are scarce. Using prediction of DNA accessibility as an example, we have illustrated the potential of transfer learning in a favorable scenario where multiple related datasets and tasks are available. The utility of transfer learning depends on how similar the new prediction task is to those of available models. Although the definition of generic measures for task similarity is an open research question, trial and error is a viable and pragmatic strategy to design transfer learning schemes because it is computationally cheap compared to exploring model architectures and parameter settings from scratch. A starting point for this search is to use models trained for tasks involving related biological processes. For example, the available models trained on in vitro transcription factor binding assays can be good initial models to train in vivo models of the same transcription factors, or models trained on different cell types of tissues. Multi-task models are particularly useful because they capture multiple biological processes, some of which might be relevant for the new task.

At the core of our contribution is an API, a unified way for software components to interact with any of these models. APIs provide modularity to software design and help to reduce code redundancy. We have demonstrated the utility of the API, which provides a generic approach both to carry out variant effect predictions and derive feature importance scores for a wide range of models. These examples are important downstream functionalities that are typically not provided by software implementations of models as provided by authors, or they may be implemented using diverse and inconsistent paradigms and interfaces. We foresee a range of future plugins that are of general use for different models. Additionally, it is straightforward to set up new instances of a Kipoi model repository. It could even be adopted in domains other than genomics because the Kipoi API is agnostic to input or output data types and machine learning frameworks.

While complying to a programmatic standard can constrain contributors and provide some initial overhead to adapting legacy software, the long-term community benefits from the standardization will outweigh short-term investments. The open software project Bioconductor and the data archive Gene Expression Omnibus are canonical examples of the expected gains. These frameworks achieve a suitable compromise between rigidly enforced structure and no structure. With this in mind, we have designed Kipoi’s API to rigorously specify specific aspects, such as providing example files to test model executability, while leaving other choices, such as the machine learning modeling framework, open to developers. We anticipate that community usage will help to develop good practices and find a reasonable balance between standardization and flexibility.

An exciting next step would be to set up open challenges for key predictive tasks in genomics with open challenge platforms, like DREAM (http://dreamchallenges.org) or CAGI (https://genomeinterpretation.org), and make the best models available in Kipoi. This would simplify and modularize the development of predictive models into three steps: first, designing training and evaluation datasets (challenge organizers); second, training the best model (challenge competitors); and third, making the model easily available for others to use (repository of trained models). Such modularization would lower the entry barrier for newcomers as well as machine learning practitioners lacking domain expertise. Moreover, as models and training datasets continue to evolve, such best-in-class models could be continuously updated and made immediately available to all. Kipoi provides important elements to this end: a standardization for data loading and model execution, nightly tests, and a central repository.

A repository of interoperable models opens the possibility of building composite models that capture how genetic variation propagates through successive biological processes. Such a sequential, modular modeling offers several advantages. First, end-to-end fitting of a complex trait such as a cellular behavior or the expression level of a gene can be too difficult because the amount of data is too scarce compared to the complexity of the phenomena. In contrast, today’s high-throughput technologies focusing on a specific subprocess offer more data at higher accuracy. For example, massively parallel reporter assays allow saturated screens in which almost the complete combinatorial sequence space can be probed for the selected molecular processes. Hence accurate models may be obtained for these elementary tasks and serve as building blocks for modeling more complex tasks. Second, modularity is a hallmark of biological processes as the same proteins are often involved in multiple processes. We therefore anticipate fruitful cross-talk between modelers sharing individual components useful for different modeling tasks. Third, such an approach would lead to models that are interpretable in terms of simpler biological processes, as opposed to black box predictors. Whether and how predictive models of elementary steps can be sequentially combined and jointly fitted to model multiple higher order biological processes of increasing complexity is an exciting research direction. Altogether, we foresee Kipoi being a catalyst in the endeavor to model complex phenotypes from genotype.

## Supplementary information

Supplementary Information Supplementary Fig. 1, Supplementary Tables 1 and 2, and Supplementary Methods

## Data Availability

All models used in this analysis are available at 10.5281/zenodo.1637796. The model configuration files in the repository link to model parameters stored in specific Zenodo digital objects and are therefore guaranteed to be reproducible and openly available. Chromatin accessibility data used for training and evaluating Divergent421 in the transfer-learning section is available at 10.5281/zenodo.2615128 in the manuscript/data/raw/tlearn directory.

## Code availability

Kipoi, kipoiseq, kipoi_veff, and kipoi_interpret are available as python packages on PyPI and their source code is available at https://github.com/kipoi/kipoi, https://github.com/kipoi/kipoiseq, https://github.com/kipoi/kipoi-veff and https://github.com/kipoi/kipoi-interpret, correspondingly. Models are hosted at https://github.com/kipoi/models. Analysis was performed with the following versions: kipoi = 0.6.4, kipoiseq = 0.2.2, kipoi_veff = 0.1.0, kipoi_interpret = 0.1.0, model repository with 5a93b7b7ae1842c35b0052e2c17afda15ec8a890 commit SHA-1 hash. Code to reproduce the results is available at https://github.com/kipoi/manuscript. Code and data are also available at 10.5281/zenodo.2615128.
