# Supplementary material for: The Kipoi repository accelerates community exchange and reuse of predictive models for genomics
Source: Nat Biotechnol. 2019 May 28;37(6):592–600. doi: 10.1038/s41587-019-0140-0 (PMC6777348; doi:10.1038/s41587-019-0140-0)
Supplement: Supplementary file 1 — Supplementary Fig. 1, Supplementary Tables 1 and 2, and Supplementary Methods [file 41587_2019_140_MOESM1_ESM.pdf]

nature biotechnology

SUPPLEMENTARY INFORMATION

In the format provided by the authors and unedited.

The Kipoi repository accelerates community exchange and reuse of predictive models for genomics

Data availability

All models used in this analysis are available at https://doi.org/10.5281/zenodo.1637796. The model configuration files in the repository link to model parameters stored in specific Zenodo digital objects and are therefore guaranteed to be reproducible and openly available. Chromatin accessibility data used for training and evaluating Divergent421 in the transfer-learning section is available at https://doi.org/10.5281/zenodo.2615128 in the manuscript/data/raw/tlearn directory.

Code availability

Kipoi, kipoiseq, kipoi_veff, and kipoi_interpret are available as python packages on PyPI and their source code is available at https://github.com/kipoi/kipoi, https://github.com/kipoi/kipoiseq, https://github.com/kipoi/kipoi-veff and https://github.com/kipoi/kipoi-interpret, correspondingly. Models are hosted at https://github.com/kipoi/models. Analysis was performed with the following versions: kipoi = 0.6.4, kipoiseq = 0.2.2, kipoi_veff = 0.1.0, kipoi_interpret = 0.1.0, model repository with 5a93b7b7ae1842c35b0052e2c17afda15ec8a890 commit SHA-1 hash. Code to reproduce the results is available at https://github.com/kipoi/manuscript. Code and data are also available at https://doi.org/10.5281/zenodo.2615128.

Editor's Note: This article has been peer-reviewed.

Žiga Avsec [1,2,12]*, Roman Kreuzhuber [3,4,12], Johnny Israeli [5], Nancy Xu [6], Jun Cheng [1,2], Avanti Shrikumar [6], Abhimanyu Banerjee [7], Daniel S. Kim [8], Thorsten Beier [9,10], Lara Urban [4,10], Anshul Kundaje [6,11]*, Oliver Stegle [4,9,10]* and Julien Gagneur [1]*

[1]Department of Informatics, Technical University of Munich, Garching, Germany. [2]Graduate School of Quantitative Biosciences (QBM), Ludwig- Maximilians-Universität München, Munich, Germany. [3]Department of Haematology, University of Cambridge, Cambridge, UK. [4]European Molecular Biology Laboratory, European Bioinformatics Institute, Hinxton, UK. [5]Biophysics Program, Stanford University, Stanford, CA, USA. [6]Department of Computer Science, Stanford University, Stanford, CA, USA. [7]Physics Department, Stanford University, Stanford, CA, USA. [8]Biomedical Informatics Program, Stanford University, Stanford, CA, USA. [9]Division for Computational Genomics & Systems Genetics, German Cancer Research Center, Heidelberg, Germany. [10]European Molecular Biology Laboratory, Genome Biology Unit, Heidelberg, Germany. [11]Department of Genetics, Stanford University, Stanford, CA, USA. [12]These authors contributed equally: Žiga Avsec, Roman Kreuzhuber.

*e-mail: avsec@in.tum.de; akundaje@stanford.edu; oliver.stegle@embl.de; gagneur@in.tum.de

Supplementary Methods

Kipoi infrastructure

Model source

Kipoi's main model repository ("model source") is hosted as a git repository at https://github.com/kipoi/models. Each folder that contains a model description file in the YAML format (`model.yaml`) is considered as a single model. The `model.yaml` specifies (i) general information including author name, publication link, license or description, (ii) required software dependencies, (iii) input-output data types, (iv) URLs to model parameters and test files, (v) default data-loader and (vi) additional information required by plugins such as variant effect prediction. In addition to the model.yaml file, further files may need to be specified. These include model.py for custom model implementations, and `dataloader.py` and `dataloader.yaml` for custom data-loader implementation and description respectively. Files required by the model like the serialized model parameters, the data-loader, and the example files are hosted on Zenodo (https://zenodo.org/) or Figshare (https://figshare.com). These external files are downloaded and validated using the MD5 hash function when the user first invokes one of the kipoi commands including `kipoi predict <model>` from the command line and `kipoi.get_model` from Python.

Multiple models of similar kind (e.g. all HOCOMOCO transcription factors) can be grouped into a model group. These can be conveniently specified using a single `mode-template.yaml` file and `models.tsv` files to reduce duplication. `models.tsv` lists all the models in the model group and their variables. A `model.yaml` for a model is created by rendering `model-template.yaml` with model's variables. One example is the CpGenie model group containing models in trained on the data from different cell-lines.

All changes to the model repository are tracked using git and the repository is regularly archived to Zenodo (https://zenodo.org/record/1637796). It is advised that contributors create a new model after substantial components of a model have been updated, ideally with version number being part of the new model name (e.g. model/<version>).

In addition to Kipoi's default model source, the user can host and seamlessly use their own, private or public, model source. Model sources are specified in Kipoi's config file and are treated equivalently to the default model source.

Depositing and testing models

New models or updates to existing models are submitted as pull requests to the Kipoi model repository https://github.com/kipoi/models. Non-source files should be deposited at the file-sharing platforms Zenodo or Figshare. We provide a quick start guide (https://kipoi.org/docs/contributing/01_Getting_started/) which includes copy-and-paste templates for all required files. These templates cover common use-cases such as DNA-sequence based models. To ensure model quality, naming guidelines scope, and appropriate specification of the distribution license, newly added models are reviewed by a member of the Kipoi core team. For each pull request, the added or updated models are automatically tested using the CircleCI continuous integration service. Additionally, one or more models selected from every model group in the repository's master branch are automatically tested every day. For each tested model, a new Conda environment with all the required dependencies will be installed, and the model will be used to obtain predictions for the example files. In order for tests to pass, no errors or warnings may be raised. The arrays returned by both, the data-loader and the model, have to be consistent with the description in their yaml files. Model predictions need to match the expected predictions. Model contributors are requested to provide information on the training dataset and specify the held-out data in the `trained_on` parameter of the model definition. Additionally, we emphasize the importance of stating the license of the contributed models to avoid copyright infringements.

The API

Kipoi's API is implemented as a python package supporting python 2.7 and python>=3.5. The package is installable from PyPI and Bioconda[23]. It provides a command line interface exposed through the `kipoi` command. Using Kipoi from the R programing language is enabled by using the `reticulate` R package. The API provides functionality necessary to manage model and data-loader dependencies, and it provides generic interfaces for executing model predictions and gradient calculations (where available). To enable generic definition of interfaces Kipoi defines two main classes: `Model` and `Dataloader`.

Model

Model is a class implementing the method `predict_on_batch(x)`. Argument x can be a single numpy array, a list of numpy arrays or a dictionary of numpy arrays. In its current version, Kipoi wraps models implemented in Keras, Tensorflow, PyTorch and Scikit-learn. For models developed in one of these frameworks, the contributor can directly provide the serialized model. A user can also deposit a custom model by implementing the Model class and hence make use of arbitrary python code or even command-line calls. For models implemented in deep learning frameworks (Keras, Tensorflow, PyTorch), the model class additionally provides two methods: `predict_activation_on_batch(x, layer, pre_nonlinearity=False)`, which returns the feature activation map of an intermediary layer (useful for transfer learning) and `input_grad(x, filter_idx=None, avg_func=None, wrt_layer=None, ...)`, which returns the gradient of the input with respect to model's predictions (useful for feature importance scores). Support for additional machine learning frameworks can be easily added.

Data-loader

The aim of the data-loader is to generate batches of data consumable by the model. It encapsulates the loading of data from input files and its pre-processing. The data-loader has to return a dictionary with three keys: inputs, targets (optional), metadata (optional). Value of the 'inputs' key is directly passed on to the model input. 'targets' provide labels useful for training or benchmarking. 'metadata' optionally provide additional information about the data samples (like sample identifier or genomic ranges of the extracted genome sequence).

To implement a data-loader, the contributor can either write a python function, generator, iterator or a Dataset class (http://kipoi.org/docs/contributing/04_Writing_dataloader.py/). Regardless of the specific implementation of the data-loader, the user will have direct access to the following methods: `batch_iter` returning batches of data stored as a dictionary with inputs, targets and metadata keys, `batch_train_iter` returning batches of data indefinitely as a tuple of inputs and targets (directly useful with the Keras' fit_generator), `batch_predict_iter` returning batches of inputs and `load_all` returning the whole dataset. Parallel data-loading is by default enabled for data-loaders written as a `Dataset` by using the DataLoader class originally implemented in PyTorch.

We provide efficient implementations of data-loaders for common sequence based models in the Kipoiseq python package (https://github.com/kipoi/kipoiseq/). Modular structure of the

package also facilities the implementation of new data-loaders by providing common transformations like genomic interval resizing or one-hot-encoding of the sequences.

Variant effect prediction and model interpretation plugins

Additional domain-specific functionality of models can be implemented in the form of additional python packages - Kipoi plugins. We implemented two plugins: variant effect prediction (https://github.com/kipoi/kipoi-veff) based on in-silico mutagenesis and model interpretation using feature importance scores (https://github.com/kipoi/kipoi-interpret).

Dependency installation

Model and the data-loader can specify dependencies installable either by the Conda package manager (https://conda.io) or the `pip` (https://pypi.org/project/pip/) package manager. Thanks to the open source efforts like conda-forge (https://conda-forge.org/) or Bioconda (https://bioconda.github.io/), the Conda package manager covers a large set of dependencies including all major bioinformatics packages. Since Conda is a package manager for any programming language, not just python, it is easy to integrate models that are implemented in another language and only expose a command-line interface. One such example is lsgkm-SVM (v0.0.1) which is precompiled and distributed through the bioconda channel. The main strength of Conda is that it can create virtual environments. This allows the user to create multiple environments for different models. To avoid the unnecessary accumulation of Conda environments, Kipoi's API allows the user to install a single environment per model group or install a 'shared' environment. We provide (and test) two shared environments which cover 19/21 model groups. Kipoi's API tracks the installed environments and can suggest the name of the installed environment for each Kipoi model via the `kipoi env get <ModelName>` command.

Docker and Singularity containers

To further simplify the usage and assure reproducibility, we provide a Singularity container[3,4] with all necessary Conda environments installed. By using the `--singularity` flag with `kipoi predict` or `kipoi veff score_variants`, the model prediction will be automatically executed in the Singularity container without the need to install any additional software dependencies except for Singularity itself (version >=2.5). Moreover, although Kipoi is not supported for the Windows operating system, a docker container for Windows with all dependencies installed is available at https://hub.docker.com/r/kipoi/models/.

Models

pwm_HOCOMOCO

Position weight matrices (PWM) for all 600 human transcription factors in HOCOMOCO v10 were downloaded from http://hocomoco10.autosome.ru/final_bundle/HUMAN/mono/HOCOMOCOv10_pcms_HUMAN_mono.txt and transformed to position specific scoring matrices (PSSM) using the pseudo-count probability of 0.001. Scanning the DNA sequences using the PSSM matrix is implemented as a Keras model consisting of a single convolutional layer with one filter whose weights are set to the PSSM, followed by global max pooling. The model operates on one-hot-encoded DNA sequence.

DeepBind

Original weights and architecture were obtained from supplementary material of the original publication[6] and were converted to Keras 2.0 models (code: https://github.com/kundajelab/DeepBindToKeras).

DeepSEA

The DeepSEA model was converted from the original Torch7 model[7] (v0.94b) to a PyTorch model using a modified version of the script https://github.com/clcarwin/convert_torch_to_pytorch. Since prediction of model tasks and variant effect prediction use different handling of reverse-complement sequences there are two models in the Kipoi model zoo dedicated to the two different use cases in order to replicate results from the original model exactly. Implementations of reverse-complement handling were taken from .lua files provided in the software package in the publication[7]. Predictions of the models and variant effects produced by the models in the Kipoi repository match the predictions produced by the website http://deepsea.princeton.edu/job/analysis/create/.

FactorNet

FactorNet models were obtained from https://github.com/uci-cbcl/FactorNet/tree/bef6f6b38e81d362162a106dc8a726ecae910138. In addition to the models available in the github repository, Daniel Quang kindly provided the trained models for CEBPB and MAFK, which were part of the internal evaluation round in the ENCODE-DREAM *in vivo*

transcription factor binding prediction challenge (http://synapse.org/encode). The models were converted to Keras 2.0 supporting the tensorflow backend.

MaxEntScan

We used MaxEntScan implemented in the maxentpy package (https://github.com/kepbod/maxentpy, v0.0.1) provided through the Bioconda channel. We implemented a data-loader that takes the reference genome FASTA file and the genome annotation GTF file as input and returns sequences of all regions [-3nt,5nt] w.r.t. the annotated 5' splice sites for the 5' model and sequences of all regions [-3nt,20nt] w.r.t. the annotated 3' splice sites for the 3' model.

HAL

The HAL model was adapted from https://github.com/Alex-Rosenberg/cell-2015/tree/ca54d1117fd28375260bfde3d1b46f3d6074f306 by implementing the identical 5' splice-site scoring function into a Kipoi's model class with the `predict_on_batch` function. Model weights were obtained from the same repository and directly applied. We implemented a data-loader that takes the reference genome FASTA file and the genome annotation GTF file as inputs and returns k-mer counts of sequences from all regions [-80nt, 80nt] w.r.t. the annotated 5' splice sites.

Labranchor

The Labranchor model was obtained from https://github.com/jpaggi/labranchor/tree/d0e232413cf39afdad7e438bef93f3cae6b816e1. The Keras model implementation provided by the authors could directly be used for the Kipoi model. We implemented a data-loader that takes the reference genome FASTA file and the genome annotation GTF file as inputs and returns one-hot-encoded sequences of all regions [-70, 0] nt relative to the annotated 3' splice sites.

Benchmarking transcription factor binding prediction models

The benchmark was scripted with Snakemake v5.3.0[30]. The complete Snakefile for the analysis described in this section is available at: https://github.com/kipoi/manuscript/blob/master/src/tf-binding/Snakefile.

Data and prediction command

The test set for transcription factor binding models was generated using 101bp contiguous intervals throughout chromosome 8 in the human genome assembly hg19. Each interval was labeled based on majority overlap with transcription factor ChIP-seq high-confidence peaks (IDR<0.05) from the ENCODE-DREAM in-vivo transcription factor binding site prediction challenge (http://synapse.org/encode). Intervals in the hg19 blacklist regions (https://www.encodeproject.org/annotations/ENCSR636HFF/) were removed. CEBPB was evaluated in the HeLa-S3 (ENCFF002CSA), JUND in HepG2 (ENCSR000EEI), MAFK in K562 (ENCFF812QPN) and NANOG in H1-hESC (ENCFF379EPK) cell type. The additional files required by FactorNet (like the DNase accessibility track) were obtained from the URLs listed in https://github.com/uci-cbcl/FactorNet/tree/bef6f6b38e81d362162a106dc8a726ecae910138/data#bigwig-files. For models that require sequence lengths of more than 101 bp, we increased the size of labeled intervals. For example, to provide 1002 bp intervals for FactorNet, we subtracted 450 bp from start coordinates and added 451 bp to end coordinates. All model predictions were obtained by running the `kipoi predict` command in the individual conda environment for each model.

Accessible-only regions

In addition to chromosome-wide evaluation, the auPRC was computed only for regions overlapping DNase-seq signal peak regions in the corresponding cell-type by more than 50% (**Supplementary Fig. 1**). DNase-seq peaks were obtained from the relaxed peaks provided by the ENCODE-DREAM *in-vivo* transcription factor binding challenge (http://synapse.org/encode).

lsgkm-SVM training

lsgkm-SVM (v0.0.1) from Bioconda (bioconda::ls-gkm=0.0.1) was used for model training and prediction. The model was retrained on ENCODE datasets using files downloaded from the same source as mentioned in the publication[31]. Preprocessing of training was performed using the gkmSVM R-package (v0.79.0) using default parameters `genNullSeqs(..., nMaxTrials=20, xfold=1, genomeVersion='hg19',..)`. For training the 322 datasets with the most peaks were chosen, similar to the lsgkm-SVM publication. Training was performed with the parameters `gkmtrain -l 11 -d 3 -c 1 -T 16 -m 5120 -v 3`. For the final model chromosome 8 and 9 were held out from training to enable model benchmarking comparable with the DeepSEA models. Trained models reached area under the receiver operating curve (auROC) similar to the original publication[31] (**Supplementary Table 1**)

Supplementary Table 1. **lsgkm-SVM training statistics** Area under the receiver operating curves (auROC) for the re-trained lsgkm-SVM models across 322 datasets along with the total number of sequences (# seqs) in the training data, the number of sequences without the nucleotide 'N' (# seqs without 'N') in the training data, and the number of sequences in the test set.

	**auROC**	**# seqs**	**# seqs without 'N'**	**# seqs test set**
Mean	0.95	22699	22698	1854
Std	0.038	13499	13499	1210
Min	0.803	6067	6067	391
1st quartile	0.931	11211	11211	861
Median	0.970	19398	19396	1424
3rd quartile	0.982	34510	34510	2878
Max	0.996	71537	71535	6342

Transfer learning

Peak File Acquisition

We downloaded DNase files for 431 biosamples (cell lines or tissues) from Roadmap (http://www.ncbi.nlm.nih.gov/geo/roadmap/epigenomics/) and ENCODE (https://www.encodeproject.org/), and processed them separately to obtain a final dataset of binary labels (0/1) indicating chromatin accessibility in each interval of the combined accessibility region for each biosample. We processed the raw data as follows: The fastq files were aligned with BWA aln (v0.7.10), where all datasets were treated as single-end. Dynamic read trimming was set to 5, the seed length was 32, and 2 mismatches maximum were allowed in mapping. After mapping, reads were filtered to remove unmapped reads and mates, non-primary alignments, reads failing platform/vendor quality checks, and PCR/optical duplicates (-F 1804). Low quality reads (MAPQ < 30) were also removed. Duplicates were marked with Picard MarkDuplicates (v1.126) and removed. The final filtered file was converted to tagAlign format (BED 3+3) using bedtools' `bamtobed` (v2.27.1). Cross-correlation scores were obtained for each file using phantompeakqualtools (v1.1).

All files with a cross-correlation quality tag below 0 were discarded. For the ENCODE data generated from the Stam Lab protocol, the datasets were trimmed to 36 bp and technical replicates were combined. After removing mitochondrial and ambiguously mapped reads, the reads were randomly subsampled to a total of 50 million reads per sample. For the ENCODE data generated from the Crawford Lab protocol, the same procedure as above was performed, except reads were trimmed to 20 bp due to the different library generation protocol. For the Roadmap data, which was all generated by the Stam Lab protocol, the same procedure as above was performed with trimming to 36 bp. Reads from multiple files were combined and subsampled to 50 million reads in case the total number of reads was more than 50 million.

These trimmed, filtered, subsampled tagAlign files were then used to generate signal tracks and call peaks. Signal tracks and peaks were called with a loose threshold ($p < 0.01$) with MACS2 (v2.1.0) to generate bigwig files (fold enrichment and p-value) and Narrow Peak files, respectively. To obtain final peak sets, we performed pseudo-replicate subsampling on the pooled reads across all replicates (taking all reads from the final tagAligns and splitting in half by random assignment to two replicates) and running IDR (v2.0.3) with a p-value threshold of <0.1 to get a consensus region set for each DNase experiment.

Data Preprocessing

We divided the genome into intervals of width 1000 bp using a stride of 200 bp. For each interval, we use the hg19 reference genome to extract the DNA sequence and assign a binary label of 0 (negative) or 1 (positive) for each of the 431 biosamples if the central 200 bp of the interval overlapped at least 50% of the accessibility IDR peak or if the accessibility IDR peak overlapped at least 50% of the central 200 bp of the interval. This resulted in the 16,551,625 intervals and 431 binary labels per interval for each of the biosamples. We use data from chromosomes 1, 8, and 21 for testing, data from chromosome 9 for validation, and the remaining data for training the models.

We selected 10 biosamples to benchmark our transfer learning procedure by performing hierarchical clustering and randomly selecting one biosample from each of the 10 clusters. Selected biosamples were: common myeloid progenitor, GM12878, Jurkat clone E61, K562, mesendoderm, mesenchymal stem cell, cardiac mesoderm, thymus, lung, and brain.

Model Architecture

We trained 3 types of models predicting chromatin accessibility given DNA sequence: one multi-task model with randomly initialized weights predicting accessibility for 421 cell-types, and two types of single-task models trained on the remaining 10 cell-types: a model with randomly initialized weights and a model with weights transferred from the multi-task model. All models were convolutional neural networks (CNN) with the BASSET[34] architecture and were implemented in Keras version 1.2 using tensorflow-gpu version 1.0.0 backend.

Transfer Learning

We used the trained multi-task model and transferred the weights from all but the final classification layer to the transferred single-task architecture. We froze the weights of all layers but the final two and replaced the final classification layer with a layer outputting a single prediction, instead of 421.

Model Training and Evaluation

Randomly initialized models and transferred models were trained using a categorical or binary cross-entropy loss, batch size of 256, epoch size of 2,500,000 and the ADAM optimizer[32] with a learning rate of 0.0003. These hyper-parameters were manually selected and were not optimized due to computational constraints. Early stopping monitoring auPRC on the validation set was used with patience of 4 epochs for models with randomly initialized weights and monitoring the validation cross-entropy loss with patience of 1 epoch for models with transferred weights. Models for individual cell types and transferred models for individual cell types were evaluated on the same test set for a given biosample (chromosomes 1, 8 and 21). For example, in the GM12878 biosample, the test set contains 135,630 positives and 2,330,052 negatives, and the validation set contains 35,526 positives and 647,116 negatives.

Predicting the molecular effects of genetic variants using interpretation plugins

The presented variants were selected from the ClinVar release from April 2018. The selection involved performing variant effect prediction for all variants in the DeepSEA model and selecting the variant with the strongest negative predicted effect in GATA2 model outputs respectively. Mutation maps centered on those two variants were generated using the mutation map commands displayed in Fig. 4d and implemented in the kipoi-veff plugin.

Predicting pathogenic splice variants by combining models

Data: ClinVar

The ClinVar release from April 2018 based on the reference genome GRCh37 was used (ftp://ftp.ncbi.nlm.nih.gov/pub/clinvar/vcf_GRCh37/clinvar_20180429.vcf.gz). Only variants in the range [-40nt, 10nt] around the splicing acceptor or variants in the range [-10, 10] nt around the splice donor of a protein coding gene (ENSEMBL GRCh37 v75 annotation) were used. The positive set comprises of variants classified as “Pathogenic” (6,310 variants) and the negative set comprises of variants classified as “Benign” (4,405 variants). Variants causing a premature stop codon were discarded. Per-variant pathogenicity/conservation scores (‘CADD_raw', 'CADD_phred', 'phyloP46way_placental', 'phyloP46way_primate’) and the dbscSNV score were obtained by VEP[40] (v92). Spidex scores were obtained from ANNOVAR (http://www.openbioinformatics.org/annovar/spidex_download_form.php).

Features

Kipoi features: For specific Kipoi models the following features were produced:

- MaxEntScan/3prime, MaxEntScan/5prime, HAL
    - <model>_ref: Model prediction for the reference allele
    - <model>_alt: Model prediction for the alternative allele
- Labranchor
    - labranchor_logit_ref: (optional) Model prediction for the reference allele on the logit scale
    - labranchor_logit_alt: (optional) Model prediction for the alternative allele on the logit scale
- All models
    - <model>_is_na: 1 if model prediction is unavailable for the variant and 0 otherwise

These features were obtained by running the `kipoi veff score_variants` command on the variants table with `-s logit_ref logit_alt ref alt logit diff` formatted as a vcf file and then parsing the returned vcf files using `kipoi_veff.parsers.KipoiVCFParser`.

dbscSNV features:

- dbscSNV_rf_score' - dbscSNV random forest score obtained with VEP
- 'dbscSNV_rf_score_isna - 1 if dbscSNV_rf_score' is unavailable for the variant and 0 otherwise

SPIDEX features:

- dpsi_max_tissue', the maximum mutation-induced change in percentage-spliced in (PSI) across 16 tissue
- 'dpsi_max_tissue_isna', 1 if dpsi_max_tissue' is unavailable for the variant and 0 otherwise
- 'dpsi_zscore', z-score transformed dpsi_max_tissue
- 'dpsi_zscore_isna, 1 if 'dpsi_zscore' is unavailable for the variant and 0 otherwise

Conservation features: All obtained using VEP

- CADD_raw, Combined Annotation–Dependent Depletion score as described in [33]
- CADD_phred, CADD phred-like rank score based on whole genome CADD raw scores
- phyloP46way_placental, phyloP (phylogenetic p-values) conservation score based on the multiple alignments of 33 placental mammal genomes including human as described in [34].
- phyloP46way_primate, phyloP (phylogenetic p-values) conservation score based on the multiple alignments of 10 primate genomes including human.

NA values were zero-imputed and each feature was standardized to have mean of zero and variance of one.

Response variable: "ClinicalSignificance" was transformed into a binary classification variable with 'Pathogenic' corresponding to class 1 and 'Benign' corresponding to class 0.

Data: dbscSNV

Table S2 from the supplementary material of [10] was used to train and evaluate models in a 10-fold cross validation (2959 variants, 1164 from the positive class).

dbscSNV features (without conservation, described in [35])

- 'PWM_ref', 'PWM_alt',
- 'MES_ref', 'MES_alt',
- 'NNSplice_ref', 'NNSplice_alt',
- 'HSF_ref', 'HSF_alt',

- 'GeneSplicer_ref', 'GeneSplicer_alt',
- 'GENSCAN_ref', 'GENSCAN_alt',
- 'NetGene2_ref', 'NetGene2_alt',
- 'SplicePredictor_ref', 'SplicePredictor_alt

Kipoi model, conservation and SPIDEX features were the same as for the ClinVar dataset.

Response variable: “Group” variable in the original table - ‘Positive’==1 and ‘Negative’==0.

Meta-model and evaluation

Logistic regression implemented in scikit-learn with default parameters was used to build the meta model using different feature subsets. 10-fold cross-validation was used (implemented in `sklearn.model_selection.cross_validate`) to evaluate models using the auROC metric.

Existing repositories of trained models

Supplementary table 2. Existing repositories of trained models, which are routinely used for benchmarking and as a starting point to rapidly develop new models in computer vision and natural language processing.

Name	Link
TensorFlow Hub	https://www.tensorflow.org/hub/
Keras-applications	https://github.com/keras-team/keras-applications
PyTorch vision models	https://pytorch.org/docs/stable/torchvision/models.html
caffee model zoo	https://github.com/BVLC/caffe/wiki/Model-Zoo

Supplementary Figures

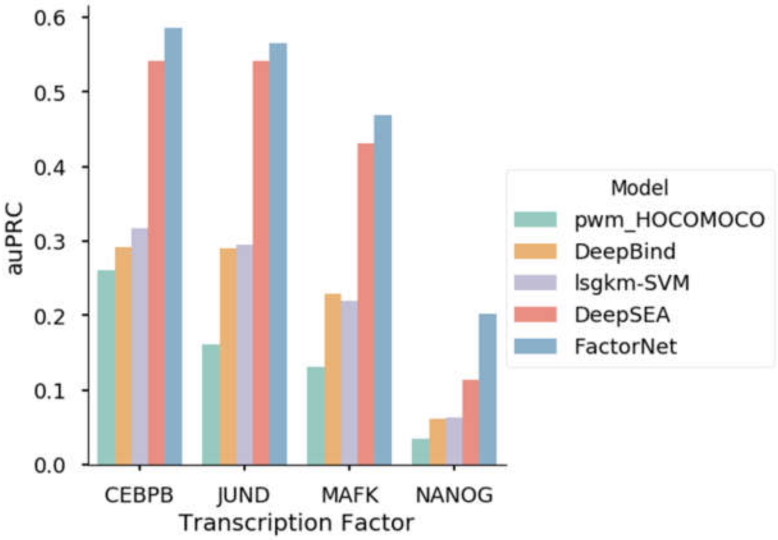

Supplementary figure 1. Performance of models highlighted in Fig. 2 for predicting ChIP-seq peaks of four transcription factors on held-out data (chromosome 8) restricted to accessible chromatin regions as measured by DNase (Methods). This shows that DeepSEA and FactorNet perform similarly when model evaluation is restricted to bound and unbound regions that strictly overlap accessible chromatin regions.

Supplementary references

30. Köster, J. & Rahmann, S. Snakemake—a scalable bioinformatics workflow engine. *Bioinformatics* **28,** 2520–2522 (2012).

31. Lee, D. LS-GKM: a new gkm-SVM for large-scale datasets. *Bioinformatics* **32,** 2196–2198 (2016).

32. Kingma, D. P. & Ba, J. Adam: A Method for Stochastic Optimization. *arXiv [cs.LG]* (2014).

33. Kircher, M. *et al.* A general framework for estimating the relative pathogenicity of human genetic variants. *Nat. Genet.* **46,** 310–315 (2014).

34. Liu, X., Wu, C., Li, C. & Boerwinkle, E. dbNSFP v3.0: A One-Stop Database of Functional Predictions and Annotations for Human Nonsynonymous and Splice-Site SNVs. *Hum. Mutat.* **37,** 235–241 (2016).

35. Jian, X., Boerwinkle, E. & Liu, X. In silico prediction of splice-altering single nucleotide variants in the human genome. *Nucleic Acids Res.* **42,** 13534–13544 (2014).
